# Assessment of some key indicators of the ecological status of an African freshwater lagoon (Lagoon Aghien, Ivory Coast)

**DOI:** 10.1371/journal.pone.0251065

**Published:** 2021-05-06

**Authors:** Mathias Koffi Ahoutou, Rosine Yao Djeha, Eric Kouamé Yao, Catherine Quiblier, Julie Niamen-Ebrottié, Sahima Hamlaoui, Kevin Tambosco, Jean-Louis Perrin, Marc Troussellier, Cécile Bernard, Luc Seguis, Marc Bouvy, Jacques Pédron, Felix Koffi Konan, Jean-François Humbert, Julien Kalpy Coulibaly

**Affiliations:** 1 Institut Pasteur d’Abidjan, Abidjan, Côte d’Ivoire; 2 Université Jean Lorougnon Guédé, Daloa, Côte d’Ivoire; 3 MNHN, UMR 7245 Molécules de Communication et Adaptation des Micro-organismes, Paris, France; 4 Université de Paris, Paris, France; 5 Laboratory of Environment and Aquatic Biology, Nangui Abrogoua University, Abidjan, Côte d’Ivoire; 6 iEES Paris, INRAE-Sorbonne Université, Paris, France; 7 HydroSciences Montpellier, IRD, Université de Montpellier, CNRS, Montpellier, France; 8 UMR MARBEC, IRD-Université de Montpellier, Montpellier, France; Cawthron Institute, NEW ZEALAND

## Abstract

The supply of drinking water is a vital challenge for the people who live on the African continent, as this continent is experiencing strong demographic growth and therefore increasing water demands. To meet these needs, surface water resources are becoming increasingly mobilized because underground resources are not always available or have already been overexploited. This situation is the case in the region of Abidjan in the Ivory Coast, where the drinking water deficit is a growing problem and it is therefore necessary to mobilize new water resources to ensure the supply of drinking water. Among the potential resources, local managers have identified a freshwater lagoon, Lagoon Aghien, That is in close proximity to the city of Abidjan. With the aim of enhancing knowledge on the ecological functioning of the lagoon and contributing to the assessment of its ability to provide drinking water, several physical and chemical parameters of the water and the phytoplankton community of the lagoon were monitored for 17 months (December 2016-April 2018) at six sampling stations. Our findings show that the lagoon is eutrophic, as evidenced by the high concentrations of total phosphorus (>140 μg L^-1^), nitrogen (1.36 mg L^-1^) and average chlorophyll-a (26 to 167 μg L^-1^) concentrations. The phytoplankton community in the lagoon is dominated by genera typical of eutrophic environments including mixotrophic genera such as *Peridinium* and by cyanobacteria such as *Cylindrospermopsis/Raphidiopsis*, *Microcystis and Dolichospermum* that can potentially produce cyanotoxins. The two rainfall peaks that occur in June and October appeared to be major events in terms of nutrient flows entering the lagoon, and the dynamics of these flows are complex. Significant differences were also found in the nutrient concentrations and to a lesser extent in the phytoplankton communities among the different stations, especially during the rainfall peaks. Overall, these results reveal that the quality of the lagoon’s water is already severely degraded, and this degradation could increase in future years due to increasing urbanization in the watershed. These results therefore raise questions about the potential use of the lagoon as a source of drinking water if measures are not taken very quickly to protect this lagoon from increasing eutrophication and other pollution sources.

## Introduction

The African continent is experiencing a rapid growth in human population [1], and the freshwater ecosystems of this continent are subjected to growing anthropogenic pressures that could potentially impact their water quality. However, except for a restricted number of large lakes mainly located in East Africa (Lake Victoria, Lake Kivu, etc.) [e.g. 2, 3], there are very limited data available on the water quality and ecological status of most lentic ecosystems in tropical Africa. From these data, it seems that growing eutrophication of these ecosystems [e.g. 4–7] and increasing pollution by pharmaceutical products [8] and other pollutants, such as organochlorine pesticides [9] and heavy metals [10], are occurring. The extent of this pollution remains largely unknown for most surface water sources. Consequently, many countries are facing a gap between the growing demand for water and the availability of knowledge regarding the water quality of potential surface water resources [11].

**Fig 1:**
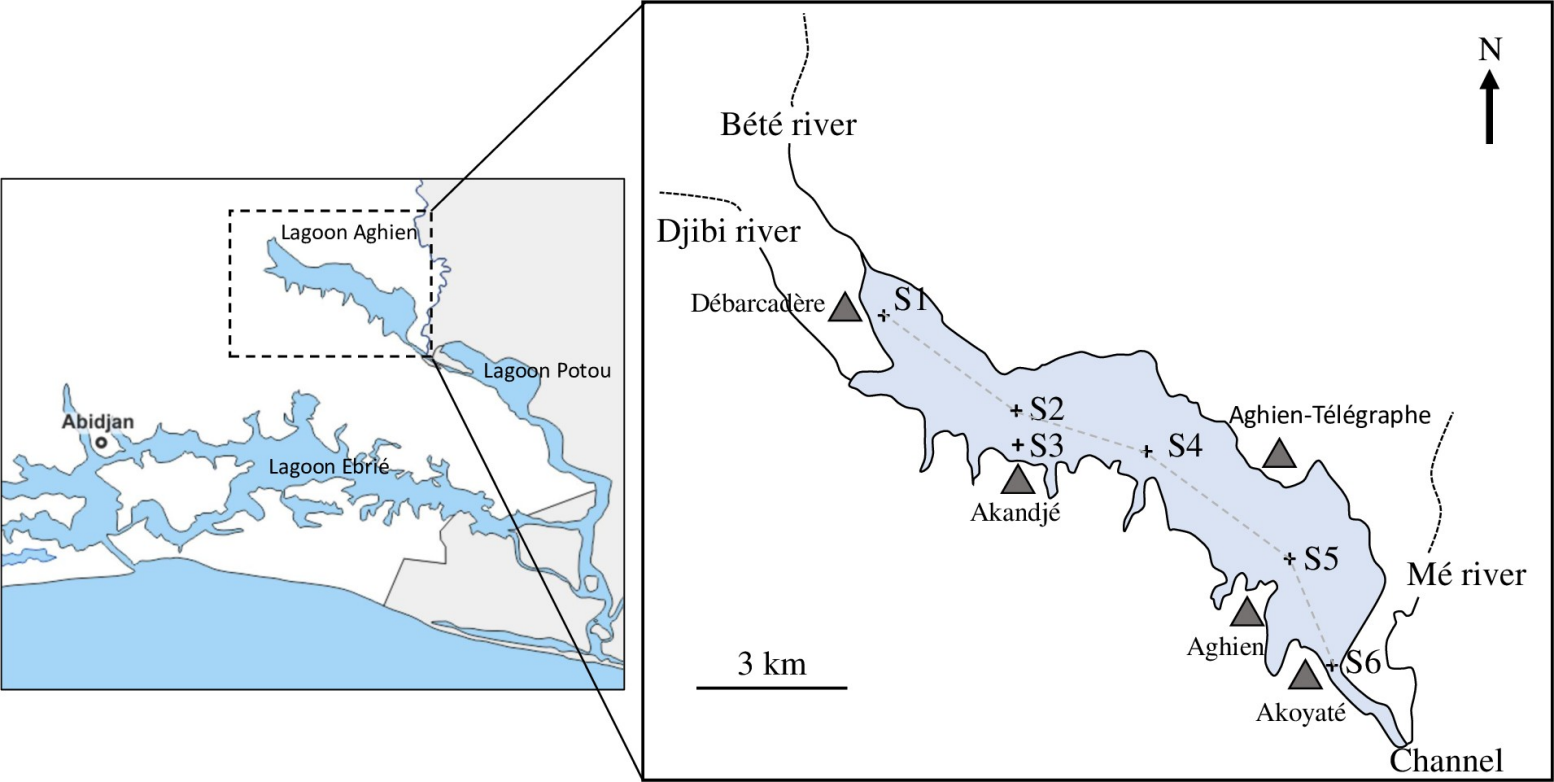
Locations of Lagoon Aghien and the six sampling stations (S1 to S6). The main villages located close to the lagoon are indicated with triangles.

In the district of Abidjan on the Ivory Coast, where the number of inhabitants is projected to rise over the next fifteen years to nearly seven and a half million people [12], water consumption is rapidly increasing. Until recently, the water supply in this urban area was based on underground pumping; however, over the last several years, the local population has experienced water shortages because the amount of groundwater extracted can no longer be increased. To better meet the water demands of the local populations, water institutions are looking for alternative surface freshwater resources, and Lagoon Aghien is a candidate. This freshwater lagoon is located in the northeastern part of the Abidjan district (Fig 1) and has until recently been used for multiple purposes by the local population living on its banks.

In the early 2010s, Ivorian water institutions (Ministère des Eaux et Forêts, MINEF, and the Office National de l’Eau Potable, ONEP) and the French Agency for Development (AFD) raised the issue of the current water quality of the lagoon in view of its use for producing drinking water. A preliminary report on the water quality of the lagoon [13] showed that the lagoon seems to be eutrophicated on the bases of its chlorophyll-a, total phosphorus (TP) and total organic carbon (TOC) concentrations. However, this report was based on a unique survey performed at eleven stations, and the results were clearly insufficient to establish an accurate assessment of the water quality and ecological status of this ecosystem. More recently, Eba *et al*. [14] identified several areas in the watershed of the lagoon that displayed various levels of vulnerability in terms of their potential contributions to pollution in the lagoon. Finally, Koffi *et al*. [15] described the global hydrological functioning of this hydrosystem and estimated the nutrient loads entering the lagoon.

However, until recently, limited data have been available on the biotic communities and ecological status of Lagoon Aghien. In particular, the phytoplankton community has been poorly investigated [16] despite (i) its importance as a primary producer for ecosystem functioning and (ii) its potential to serve as an integrated assessment of the ecological/trophic status of the lagoon [17]. In this global context, a 17 months monitoring survey of the physical, chemical and biological variables in the lagoon was performed. This study aimed to contribute to the assessment of the ecological status of Lagoon Aghien and, consequently, to the evaluation of the current capacity of the lagoon to provide drinking water for people living in the peri-urban area of Abidjan.

## Material and methods

### Study site: Lagoon Aghien

Lagoon Aghien (5°22’N to 5°26’N and 3°49’W and 3°55’W) is a freshwater lagoon located in the district of Abidjan in the south of the Ivory Coast, approximately 5 km north of the city of Bingerville (Fig 1). The climate of this area is a typical tropical monsoon climate that is characterized by two rainy seasons (April/May–June and October–November) separated by two dry periods (July–September and December–March).

Lagoon Aghien covers a surface area of 19.5 km² with a perimeter of 40.72 km, a volume of 75 km^3^ and a maximum depth of 10 m [15]. Lagoon Aghien is connected with Lagoon Potou by a 5-km-long channel. Together with the brackish Lagoon Ebrié, these lagoons form the largest lagoon system in the Ivory Coast and in West Africa [18]. In contrast with the other lagoons composing this large system, Lagoon Aghien transitioned to a freshwater ecosystem after the permanent closure of the mouth of the Comoé River due to the construction of the Vridi canal; this closure limited the effects of tides and saltwater incursions entering Lagoon Aghien [19]. Nevertheless, the lagoon is still under tidal influence near the channel that connects Lagoons Aghien with Lagoon Potou inducing flow direction inversions twice a day [15]. Finally, three rivers (Bété, Djibi and Mé) contribute to the filling of Lagoon Aghien.

The total surface area of the watershed of Lagoon Aghien is 4,416 km^2^, and the respective areas of the Bété, Djibi and Mé Rivers watersheds are 205, 85 and 4,126 km^2^ [20]. The Mé and Bété catchments mainly include natural and agricultural lands, whereas the Djibi catchment contains mostly urbanized areas. The northern part of the watershed close to the lagoon consists of hevea and oil palm plantations. The southern part of the watershed is strongly influenced by urban activities, as shown by the recent deforestation of this region for the construction of settlements. Finally, several villages are located on the shores of the lagoon, together comprising >10,000 inhabitants, and the lagoon is used for multiple purposes by the people living in these villages, including washing dishes and clothes, bathing, fishing and consumption [21].

### Sampling strategy

Monthly sampling was performed from December 2016 to April 2018 at six sampling stations. As shown in Fig 1, five sampling stations (S1, S2, S4, S5 and S6) were located on a transect oriented along the main NW-SE axis of the lagoon. One additional sampling station (S3) was located close to the shoreline area of Akandjé village to determine the eventual direct impacts of the activities of the local populations on the water quality. The locations and depths of the sampling stations are reported in S1 Table.

At each sampling station, the temperature, turbidity, dissolved oxygen and chlorophyll-a concentrations were recorded along the water column by using a YSI EXO2 multiparameter probe. Monthly rainfall values were obtained from the averages of the data collected at two weather stations (Anyama and Atchokoi). For the nutrient and phytoplankton analyses, water samples were collected in the first meter of the water column by using an integrated sampler according to the methods outlined in Laplace-Treyture *et al*. [22]. The collected water samples were transferred to two clean bottles that were stored in a cool box until they were returned to the laboratory. The phytoplankton samples were fixed in a formaldehyde solution (5% in final concentration). In addition, phytoplankton community sampling was performed by using a 20 μm mesh plankton net to assist with the qualitative assessment of this community.

### Chlorophyll-a estimation

During the monitoring period, chlorophyll-a (Chl-a) concentrations were recorded in the water column by using the YSI EXO2 multiparameter probe. As we found the Chl-a concentrations provided by the probe to be underestimated, we used another dataset collected during mesocosm experiments performed in Lagoon Aghien in February 2018 (paper in preparation) to correct these data. As shown in S2 Table, estimations of the Chl-a concentrations were performed for the same samples by using the YSI probe and after the pigment extractions and spectrophotometric measurement. A highly significant correlation (R^2^ = 0.78; p<0.001) was observed between these two estimates and we obtained the following simple linear regression: Chl-a _probe_ = Chl-a _spectro_ * 0.1445 + 2.9809. Then we used this equation to correct the values provided by the probe during our lagoon monitoring survey and used these estimated values (Chl-a _est_ = (Chl-a _probe_− 2.9809) / 0.1445) in all our subsequent analyses.

### Nutrient analyses

All the nutrient analyses were performed following the instructions of the LCK cuvette test systems provided by of the manufacturer (Hach^®^ Company). The preparation of samples for the dissolved nutrient analyses was performed using LCK cuvette test systems LCK 304, 341, 339 and 349 for ammonium (NH_4_), nitrites (NO_2_), nitrates (NO_3_) and soluble reactive phosphorus (SRP) respectively, after the water samples were filtered through nylon membranes (Whatman^TM^, porosity 0.45 μm, diameter 47 mm). The preparation of sample for the total nitrogen (TN) and total phosphorus (TP) analyses was performed using unfiltered water samples, with LCK cuvette test systems LCK 138 and 349, respectively. The protocols for the TN and TP measurments include a digestion step at a high temperature, performed with a HACH HT 2500 thermostat. Finally, colorimetric determinations of the concentrations were made for all the nutrients using a HACH DR6000 UV VIS spectrophotometer.

### Phytoplankton analyses

The identification and counting of phytoplankton genera were performed on an inverted microscope (Nikon Eclipse TS100) using the Utermöhl method [23], according to the AFNOR 15204 standard. For each sample, a minimum of 30 fields of view, selected at random and distributed over the entire surface of the bottom of counting chamber, were observed under the microscope such that at least 500 counting units (cells, colonies or filaments/trichomes) were counted. Except for some cyanobacteria, the cells were directly counted,. For *Microcystis*, two categories of colonies were considered corresponding to their diameters (< or > 200 μm). The mean number of cells per category was estimated under an upright microscope by counting the number of cells in 30 colonies per category after the colonies were gently spread between slides and coverslips. For filamentous cyanobacteria (*Aphanizomenon*, *Cylindrospermopsis/Raphidiopsis*, *Limnothrix*, *Lyngbya*, *Oscillatoria*, *Planktothrix* and *Pseudanabaena*), filament lengths of 100 μm were considered, and the mean cell number per 100-μm of filament was estimated on 30 filaments per genus according to the method described by Catherine *et al*. [24]. Finally, for all genera, the results of the cell counts were expressed as numbers of cells per milliliter. The mean cell biovolumes were estimated for nine abundant genera (*Microcystis*, *Cylindrospermopsis/Raphidiopsis*, *Limnothrix*, *Oscillatoria*, *Dolichospermum*, *Scenedesmus*, *Staurastrum*, *Aulacoseira* and *Peridinium*) using measurements of 30 cells per genus. For all the other genera, we used the standard cell biovolume values provided in the HELCOM PEG Biovolume reports (https://helcom.fi/helcom-at-work/projects/peg/).

Finally, a functional classification of the dominant genera in the phytoplankton community of the Lagoon Aghien was performed according to the paper by Reynolds *et al*. [25] while taking into account the recommendations of Padisak *et al*. [26].

### Statistical analyses

The Shannon-Weaver index was calculated at the genus level using the obtained biovolume values by with PAST software (V3.24) [27]. The ß-diversity was estimated on the biovolume values of the genera (i) each month by comparing the diversity of the phytoplankton community among each sampling station (spatial dimension) and (ii) over the whole monitoring period by comparing the diversity values of the phytoplankton community among the 17 sampling dates (temporal dimension). The ß-diversity analysis was performed using the betapart package in R [28]. A standard normal homogeneity test was used to detect both abrupt and gradual linear trend homogeneity breaks with XLStat (V2.01) software. Multivariate analyses (principal component analysis (PCA) and canonical correspondence analysis (CCA)) were performed using XLStat (V2.01) software.

## Results

### Spatiotemporal variations in physical and chemical variables

As expected under tropical climate conditions, strong rainfall variations were found during the monitoring period (Table 1), and the two rainfall peaks occurred in June and October 2017. The lowest water temperatures were recorded during the period between the two rainy seasons (July and August 2017), with temperatures ranging between 26.3 and 31.6°C during the survey. A large variation range was observed in the monthly average turbidity values (from 7.2 to 198 NTU). The highest turbidity values were recorded in May, July and September 2017. All the data collected with the YSI probe are available in S3 Table.

**Table 1:** Monthly average values (± standard deviations) of the chemical and physical variables measured at the six sampling stations in Lagoon Aghien.

	TN (mg N L^-1^)	Ammonia (mg N-NH_4_ L^-1^)	Nitrite (mg N-NO_2_ L^-1^)	Nitrate (mg N-NO_3_ L^-1^)	TP (mg P L^-1^)	SRP (mg P-PO_4_ L^-1^)	TN/TP	Turbidity (NTU)	Rainfall (mm)	Water Temp. (°C)	Chl-a (μg L^-1^)
**Dec-16**	1.73 ±0.5	0.46 ±0.15	0.05 ±0.00	0.38 ±0.08	0.15 ±0.01	0.07 ±0.02	11±3	ND	98	ND	ND
**Jan-17**	2.18 ±0.9	0.11 ±0.12	0.03 ±0.01	0.29 ±0.08	0.15 ±0.01	0.08 ±0.01	14±6	7.24±2.4	19	30.2±0.3	127±37
**Feb-17**	2.06 ±0.6	0.07 ±0.03	0.08 ±0.01	0.41 ±0.02	0.16 ±0.02	0.08 ±0.07	13±3	10.34±4.2	54	31.0±0.6	167±84
**Mar-17**	1.36 ±0.4	0.08 ±0.02	0.08 ±0.01	0.43 ±0.05	0.16 ±0.01	0.08 ±0.03	8±2	13.20±6.9	28	31.6±0.8	129±62
**Apr-17**	1.58 ±0.5	0.09 ±0.02	0.07 ±0.01	0.40 ±0.09	0.17 ±0.01	0.12 ±0.02	9±2	24.8±21.0	136	31.3±0.4	86±24
**May-17**	1.73 ±0.3	0.09 ±0.02	0.08 ±0.03	0.40 ±0.04	0.26 ±0.08	0.06 ±0.01	7±2	121.7±103.5	104	29.8±0.3	89±10
**Jun-17**	2.94 ±0.3	0.19 ±0.04	0.06 ±0.01	0.66 ±0.07	0.56 ±0.36	0.12 ±0.05	8±7	85.2±32.4	566	28.0±0.2	43±19
**Jul-17**	1.52 ±0.4	0.23 ±0.10	0.05 ±0.00	1.03 ±0.15	0.16 ±0.03	0.08 ±0.01	10±2	198.0±239.3	150	26.4±0.3	26±12
**Aug-17**	1.59 ±0.4	0.12 ±0.05	0.05 ±0.00	1.06 ±0.31	0.21 ±0.08	0.08 ±0.01	9±4	38.4±8.9	72	26.3±0.2	66±38
**Sep-17**	2.00 ±0.6	0.05 ±0.04	0.04 ±0.00	0.51 ±0.05	0.23 ±0.06	0.07 ±0.01	10±5	109.0±154.2	115	28.1±0.7	48±12
**Oct-17**	2.62 ±0.3	0.09 ±0.02	0.04 ±0.00	0.47 ±0.11	0.47 ±0.30	0.12 ±0.02	8±5	29.1±11.4	386	29.6±0.8	61±28
**Nov-17**	1.91 ±0.1	0.07 ±0.03	0.04 ±0.00	0.57 ±0.23	0.16 ±0.01	0.08 ±0.07	12±2	24.2±17.0	213	29.6±1.7	35±9
**Dec-17**	1.52 ±0.5	0.07 ±0.05	0.04 ±0.00	0.44 ±0.04	0.14 ±0.01	0.04 ±0.02	11±4	28.5±27.1	54	28.4±0.6	32±11
**Jan-18**	2.05 ±0.6	0.05 ±0.03	0.05 ±0.00	0.49 ±0.10	0.15 ±0.02	0.07 ±0.04	14±3	12.1±8.6	35	29.6±0.8	42±23
**Feb-18**	1.91 ±0.2	0.08 ±0.02	0.05 ±0.00	0.53 ±0.06	0.22 ±0.04	0.08 ±0.01	9±1	14.1±4.8	75	29.7±0.7	59±13
**Mar-18**	1.56 ±0.5	0.03 ±0.02	0.01 ±0.00	0.32 ±0.06	0.22 ±0.04	0.05 ±0.01	7±2	13.8±7.3	148	30.7±0.8	46±17
**Apr-18**	1.80 ±0.3	0.09 ±0.02	0.05 ±0.02	0.51 ±0.16	0.36 ±0.18	0.10 ±0.03	7±4	22.1±15.0	89	30.8±0.3	43±17

TN = total nitrogen; TP = total phosphorus; SRP = soluble reactive phosphorus; Chl-a = chlorophyll-a

**Fig 2:**
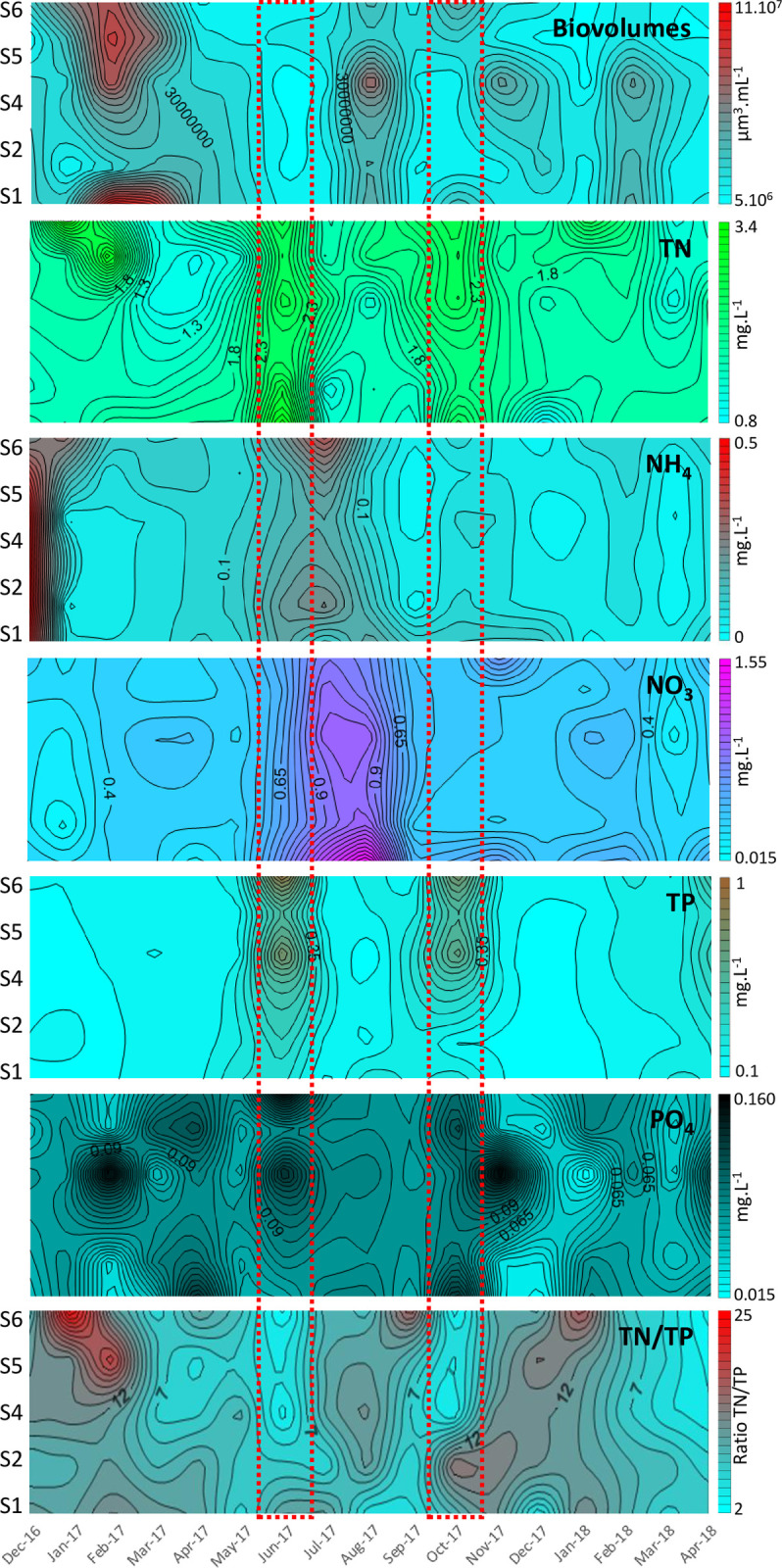
Spatiotemporal variations in the total biovolumes of the phytoplankton community and in the nutrient concentrations obtained during the monitoring of Lagoon Aghien. Only the sampling points located on the transect (S1, S2, S4, S5, and S6) are displayed in this figure. S1, S2, S4, S5, and S6 indicate the sampling stations; Biovolumes represents the phytoplankton biovolumes; TN represents total nitrogen; TP indicates total phosphorus; and N/P represents the TN/TP ratio. The red dotted rectangles correspond to the two rainfall peaks.

All the nutrient concentration data obtained in this study are available in S4 Table. The TP concentrations ranged between 0.14 ±0.01 and 0.56 ±0.36 mg P L^-1^, and the highest values were recorded during the two rainfall peaks in June and October 2017 (Table 1). A positive correlation was obtained between the monthly TP concentrations and rainfall values (R^2^ = 0.7, p<0.0001, n = 17). At the spatial scale, large variations occurred in the TP concentrations during the rainfall peaks (June and October 2017), as shown by the largest standard deviation values recorded in the TP concentrations (Table 1). The highest TP concentrations were found at sampling stations 5 and 6 (Fig 2). Finally, the highest SRP concentrations were found during the two rainfall peaks.

Concerning nitrogen, the TN concentrations ranged between 1.52 ±0.4 and 2.94 ±0.3 mg N L^-1^ and were positively correlated with the monthly rainfall values (R^2^ = 0.5, p<0.001; n = 17). In contrast with TP, the TN concentrations were homogeneous among the six sampling stations during the rainfall peaks (Fig 2). However, when looking at the data available for sampling station 3, which is located away from the transect and close to the bank, the TN concentrations appeared to be higher at this station than at sampling stations 2 and 4 (S1 Fig) (Friedman test, p<0.05; n = 17); these two stations were located closer to station 3 than the other stations but were more distant from the banks of the lagoon that station 3 was (Fig 1).

Interestingly, the increase in ammonium concentrations measured in July 2017, just after the first rainfall peak, and the decrease in ammonium concentrations and the increase in nitrate concentrations measured in August 2017 suggest the potential occurrences of ammonification and nitrification processes in the lagoon during this period (Fig 2).

The TN/TP ratios were always ≥7, and the highest the TN/TP ratio values were associated with the highest measured phytoplankton biovolume values. However, the lowest TN/TP ratios were found during the rainfall periods, potentially supporting that the P loads were proportionally higher than the N loads during these periods (Table 1).

Water temperature and oxygen concentration variations were found in the water column of Lagoon Aghien (S2 Fig). A temperature gradient was found in the water column during the survey; the temperature difference between the top and the bottom of the water column was always <3°C. In addition, significant decreases in the dissolved oxygen concentrations sometimes led to hypoxia or even anoxia at the bottom of the lake, especially at the two deepest sampling stations (Stations 2 and 4). At these stations, anoxia was observed during the warmest period from January to April when the highest phytoplanktonic biovolumes were observed (Table 1 and Fig 2).

### Spatiotemporal variations in the structure and biovolume of the phytoplankton community

#### Spatial and temporal variations in the biovolumes of the phytoplankton community

**Fig 3:**
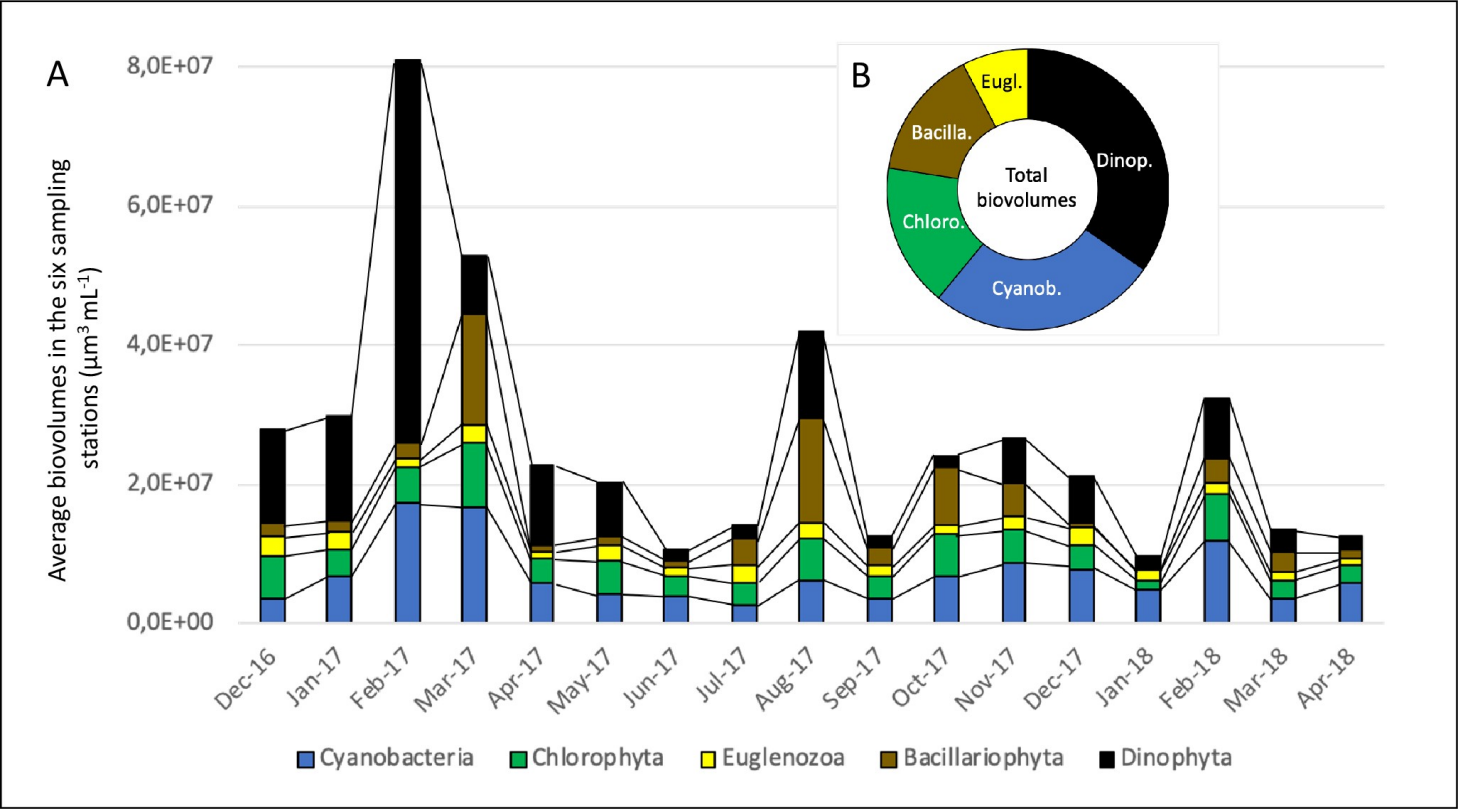
Monthly variations in the average biovolumes of the five main phytoplankton groups during the monitoring of Lagoon Aghien (A) and the average relative contributions of the five main phytoplankton groups to the total phytoplankton biovolume (B).

All the biovolume data are available in S5 Table. As shown in Figs 2 and 3A, the highest biovolume values were found during the dry season, particularly from February-March 2017 and in August 2017 and in February 2018. A comparison between the two major dry periods (December-April) in 2016/17 and in 2017/18 also indicated that there was a marked difference in the biovolumes measured at these times, with the highest values being recorded in 2016/17 (Sign Test, P < 0.0001; n = 5).

Large variations were found in the spatial distribution of the phytoplankton biovolumes in the lagoon depending on the sampling date. The Wilcoxon signed-rank test results showed that significant differences were found only between station 1 and stations 2, 5 and 6 (p<0.05; n = 17).

#### Structural and functional diversity of the phytoplankton community in Lagoon Aghien

At the scale of the main phytoplankton groups (Fig 3B), the total biovolume of the phytoplankton community in Lagoon Aghien during our monitoring period was distributed among Dinophyceae (34.6% of the total biovolumes), Cyanobacteria (26.3%), Chlorophyta (16.5%), Bacillariophyta (15.0%) and Euglenozoa (7.6%). As shown in Fig 3A, the peaks in the overall biovolume were mainly associated with increases in the biovolumes of Dinophyta, Cyanobacteria and, to a lesser extent, Bacillariophyta.

As shown in S6 Table, 52 genera were identified in the phytoplankton community during the course of the study (22 Chlorophyta genera; 15 Cyanobacteria; 9 Bacillariophyta; 5 Euglenozoa and 1 Dinophyta). Among them, 18 were found on all sampling dates at least at one sampling station. Nineteen other genera were found on more than half of the sampling dates. In terms of their presence at each sampling date and their average biovolume, 16 genera dominated the community and represented >95% of the total biovolume of the phytoplanktonic community: (i) six of these genera belong to Cyanobacteria: *Cylindrospermopsis/Raphidiopsis* and *Dolichospermum* (two nitrogen-fixing Cyanobacteria genera), *Oscillatoria*, *Limnothrix*, *Planktothrix* and *Microcystis*, all of which being susceptible to produce cyanotoxins; (ii) four of these genera belong to Chlorophyta: *Pediastrum*, *Closterium*, *Staurastrum* and *Scenedesmus*; (iii) three belong to Bacillariophyta: *Aulacoseira*, *Melosira* and *Cyclotella*; and (iv) two belong to Euglenozoa: *Trachelomonas* and *Euglena*. *Peridinium*, which displayed the highest biovolume values, was the only genus representing Dinophyta.

To obtain a functional classification of the dominant phytoplankton genera growing in Lagoon Aghien, an assignation of these genera into functional groups was performed according to the functional classification of Reynolds *et al*. [25] updated by Padisak *et al*. [26]. Among the 16 dominant genera representing >95% of the total phytoplankton biovolume measured in Lagoon Aghien, three genera (*Aulacoseira*, *Oscillatoria* and *Cyclotella*) were not assigned to a functional group because the species belonging to these three genera were classified into different functional groups. The 13 other genera belonged to nine functional groups, and these groups are typical of the warm mixed layer of eutrophicated shallow lakes (Table 2). Moreover, most of these genera display tolerances to light deficiency and two of the genera display tolerances to nitrogen deficiency (the nitrogen-fixing Cyanobacteria genera). Finally, when looking at the nutritional behavior of these dominant genera, three of these genera, together representing more than 40% of the total phytoplankton biomass (*Peridinium*, *Trachelomonas* and *Euglena*), are known to be mixotrophic, while all the other genera are phototrophic.

**Table 2:** Functional classification of the dominant genera in the phytoplankton community of Lagoon Aghien.

Phytoplankton genera (% of the total phytoplankton biovolume)	Functionnal group	Habitat templates	Tolerances	Source of energy and carbon
*Peridinium* (34.6%)	L_O_	Deep and shallow, oligo to eutrophic, medium to large lakes	Segregated nutrients	Mixotrophic
*Cylindropsermopsis*/ *Raphidiopsis* (8.3%)	S_N_	Warm mixed environments	Light-, nitrogen- deficient	Phototrophic (N_2_ fixing)
*Pediastrum* (8.1%) *Scenedesmus* (0.5%)	J	Shallow, warm mixed, enriched systems		Phototrophic
*Aulacoseira* (7.5%)	-*			Phototrophic
*Oscillatoria* (6.2%)	-*			Phototrophic
*Melosira* (6.1%)			Mild light and carbon deficiency	Phototrophic
*Staurastrum* (1.4%)	P	Eutrophic epilimnia		
*Closterium* (4.4%)				
*Trachelomonas* (6.1%)	W2	Meso-eutrophic shallow lakes and ponds		Mixotrophic
*Limnothrix* (5.3%)	S1	Turbid mixed environements, shade-adapted cyanobacteria	Highly light deficiency	Phototrophic
*Planktothrix* (0.7%)				
*Microcystis* (3.8%)	M	Mixed layer of eutrophic to hypertrophic lakes at low latitudes	Hight insolation	Phototrophic
*Dolichospermum* (1.0%)	H1/H2	Mesotrophic to eutrophic shallow lakes	Low nitrogen and carbon	Phototrophic (N_2_ fixing)
*Euglena* (0.8%)	W1	Ponds, even temporary, rich in organic matter from husbandry or sewages	High BOD	Mixotrophic
*Cyclotella* (0.6%)	-*			Phototrophic

* Not possible to assign a functional group at the genus level

#### Spatiotemporal variations in the α- and ß-diversity of the phytoplankton community

The mean number of genera (S) found at the different sampling stations on each sampling date was 22.3±4.5, while the average values of the Shannon index calculated at the genus level were close to 2 (H = 1.98±0.37). As shown in S3 Fig, no marked trend was found in the variation in the richness or diversity of the phytoplankton community during the survey. However, a standard normal homogeneity test (SNHT) performed to detect changepoints in the Shannon values showed that a significant difference (p = 0.009; n = 102) existed between the Shannon values estimated during the first three sampling months (December 2016 to February 2017) and those recorded during the other sampling dates, meaning that the diversity of the phytoplankton community was lower when the biovolume values were very high.

**Fig 4:**
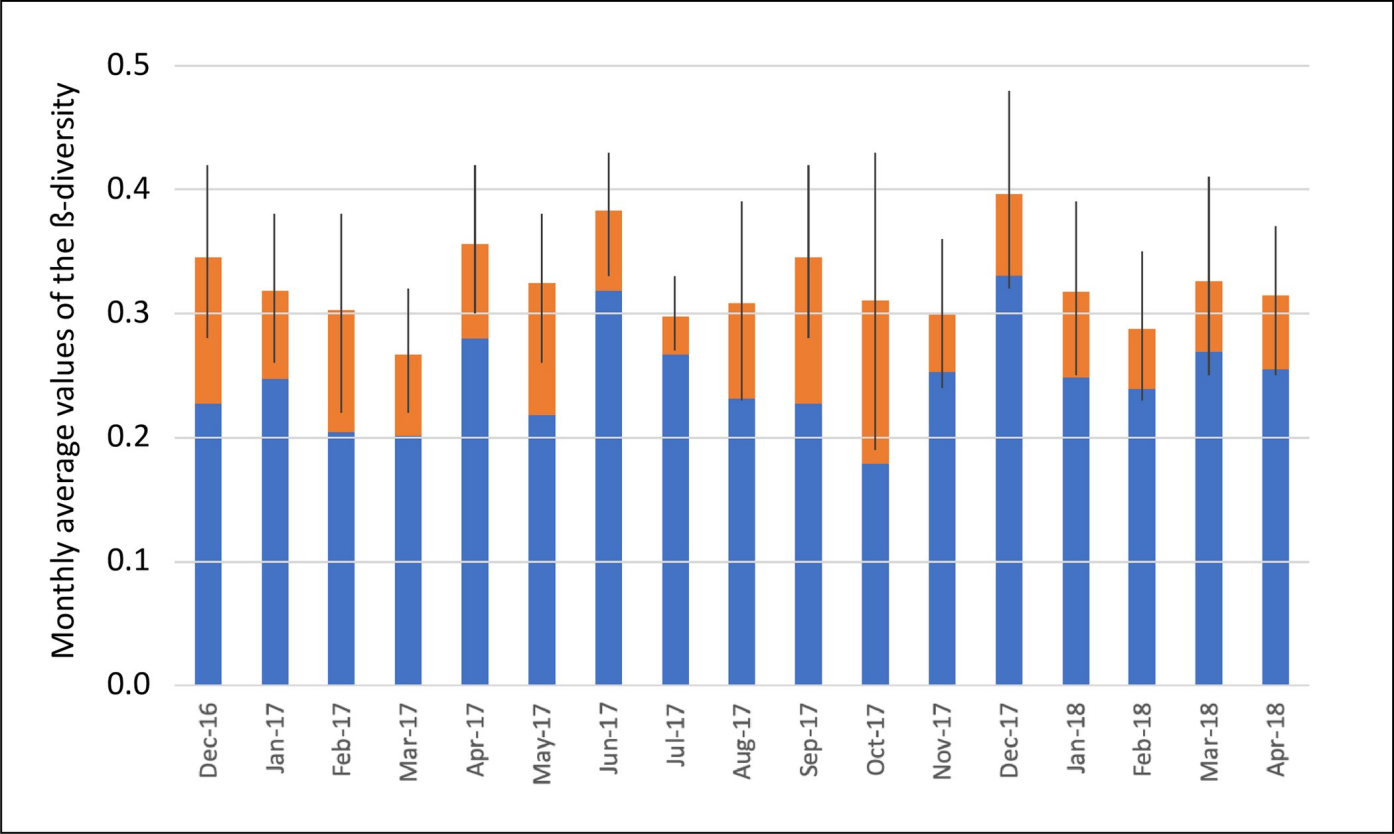
Variations in the average monthly ß-diversity values at the six sampling stations during the 17-month monitoring period. The blue bar indicates the turnover component of the ß-diversity; the orange bar represents the nestedness component of the ß-diversity; and the black line indicates the standard deviation around the monthly average value of the total ß-diversity.

As shown in Fig 4, the average of the total ß-diversity values estimated each month at the six sampling stations ranged from 0.27 to 0.38, with an average value equal to 0.32. These overall ß-diversity values were always <0.4, suggesting that there was global stability in the structure of the phytoplankton community at the genus taxonomic level at the six sampling stations in Lagoon Aghien. When looking at the two components of the ß-diversity (Fig 4), the turnover component was on average almost four times greater than the nestedness component. This result means that the changes that occurred in the phytoplankton community among the sampling stations were mainly due to the replacement of some genera by other genera.

Finally, when looking at the temporal dimension of ß-diversity, the average value was 0.15, while the minimum and maximum values were 0.08 and 0.24, respectively. This finding means that the structure of the phytoplankton community throughout the whole lagoon, as estimated on the basis of the biovolumes of each genus, was very stable during the monitoring period.

#### Relationship among the physical, chemical and phytoplankton variables in Lagoon Aghien

Principal component analysis (PCA) was performed on the physical and chemical variables and on the Chl-a values recorded during this study at the six sampling stations (S4 Fig). The inertia values of the first three axes were 25.0%, 18.2% and 11.8%. The variables that mainly contributed to the first three axes were NH_4_, rainfall, water temperature, NO_3_ and total phosphorus (19.2%, 19.0%, 15.9%, 13.3% and 11.4%, respectively) for axis 1; NO_3_, total nitrogen, water temperature, and total phosphorus (16.5%, 15.5%, 13.3% and 12.8%, respectively) for axis 2; and NO_2_, Chl-a, and dissolved oxygen (42.5%, 25.9%, and 11.5%, respectively) for axis 3.

As expected, rainfall appeared to be a major driver of the physicochemical and biological functioning of Lagoon Aghien; this variable was positively correlated (p<0.05) with total phosphorus (Pearson’s r = 0.6) and total nitrogen (Pearson’s r = 0.5) and negatively correlated (p<0.05) with water temperature (Pearson’s r = -0.22; p<0.05). The NH_4_ and NO_3_ concentrations were both negatively correlated (p < 0.05) with water temperature (Pearson’s r = -0.42 and r = -0.72, respectively). No significant correlations were observed between Chl-a and any of the other variables included in this analysis. The first factorial plane clearly showed that the largest variations among the sampling stations on each sampling date were recorded in June and July 2017 and, to a lesser extent, in October 2017. On these three sampling dates, the values measured at station 6 were the most differentiated from those measured at the other stations.

**Fig 5:**
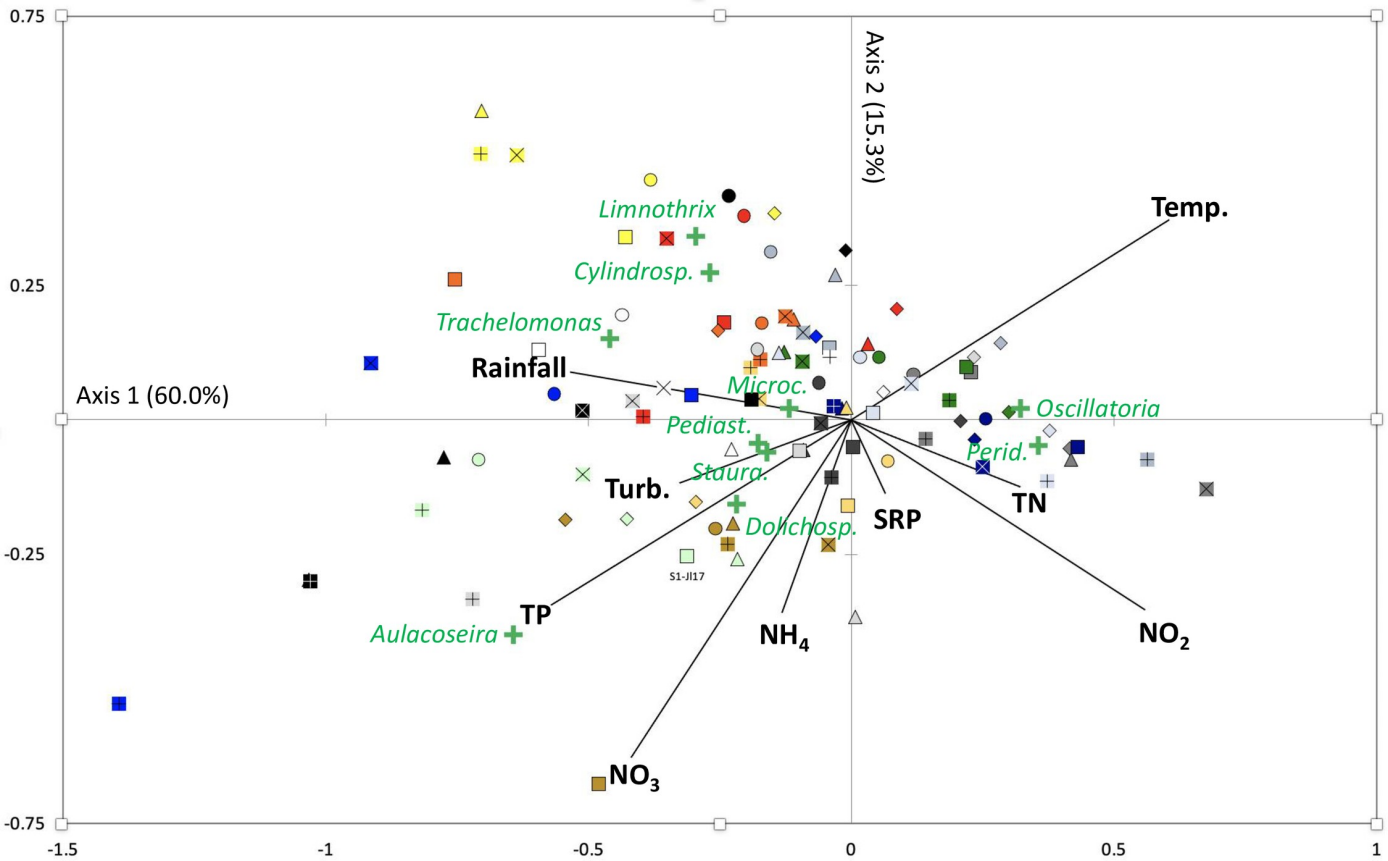
Canonical correspondence analysis was performed on the measured physical and chemical variable values and on the biovolumes of the 10 dominant genera in the phytoplankton community during the monitoring of Lagoon Aghien from January 2017 to April 2018. Cylindrosp. = *Cylindrospermopsis*; Microc. = *Microcystis*; Pediast. = *Pediastrum*; Staura. = *Staurastrum*; Dolichosp. = *Dolichospermum*; and Perid. = *Peridinium*. SRP = soluble reactive phosphorus; TN = total nitrogen; TP = total phosphorus; NH_4_ = ammonium; NO_2_ = nitrite; NO_3_ = nitrate; Turb. = turbidity; Temp. = water temperature. Significance of the symbols: Square symbol = station 1; circle = station 2; diamond = station 3; triangle = station 4; “x” = Station 5; “+” = station 6. Colors of the symbols: Jan 2017 = blue gray; Feb 17 = gray 2; Mar 17 = dark blue; Apr 17 = green; May 17 = gold; Jun 17 = blue; Jul 17 = light green; Aug 17 = brown; Sep 17 = white; Oct 17 = black; Nov 17 = red; Dec 17 = orange; Jan 18 = light blue; Feb 18 = gray 3; Mar 18 = yellow; Apr 18 = gray 1.

Canonical correspondence analysis (CCA) was performed on the physical and chemical variables measured in Lagoon Aghien and on the biovolume values of the ten dominant phytoplanktonic genera (containing >90% of the total phytoplankton biovolume) (Fig 5). As shown by the total inertia partitioning between the constrained and unconstrained CCA results, the variations in the measured physical and chemical data only explain a small portion (22%) of the variations in the biovolume values of the ten dominant phytoplankton genera. The main contributors to the three first axes were as follows: (i) for the first axis (inertia = 60.0%), *Peridinium* (contribution = 39%), *Aulacoseira* and *Trachelomonas* (contributions = 29.6% and 11.5%, respectively) were the main contributors, (ii) for the second axis (inertia = 15.3%), *Aulacoseira* (contribution = 44.8%) and *Cylindropsermopsis* and *Limnothrix* (F2 contributions = 22.9% and 22.8%, respectively) were the main contributors, and (iii) for the third axis (inertia = 10.5%), *Trachelomonas* (contribution = 32.3%), *Aulacoseira* (contribution = 22.2%) and *Oscillatoria* (contribution = 19.5%) were the main contributors. *Peridinium* was clearly associated with the dry and hot periods (Dec 2016-February 2017), a ruslt that contrasted with all other genera. The position of *Aulacoseira* in the projection is explained by its high biovolume values recorded at sampling station 6 in March, August and October 2017 and in April 2018.

## Discussion

### Dynamics and origins of nutrients

Our data provided interesting insights regarding nutrient availability in the lagoon and on the potential sources of these nutrients.

First, the high TN and TP concentrations recorded during the rainfall peaks (June and October 2017) at the sampling stations (1, 2, 5, and 6) located near the mouths of the three main tributaries (the Djibi, Bété and Mé rivers), suggest that rainfall events seem to play a major role in the nutrient loads in the lagoon. This hypothesis is supported by the paper by Koffi *et al*. [15], who showed that high TN and TP fluxes were recorded in the Djibi, Bété and Mé rivers during rainy periods. The low TN/TP ratios (approximately 7–8) recorded during rainfall periods when compared to those recorded during the rest of the years (up to approximately 14) suggest that the P loads are proportionally higher than the N loads during these periods. As the watersheds of the Bété and Mé rivers are mainly composed of natural and agricultural lands, proportionally high N inputs into the lagoon are expected from these rivers. However, as shown by Chianu *et al*. [29] and Masso *et al*. [30], the use of N fertilizers is generally very low in Sub-Saharan Africa, leading to a strong, negative N balance in soils in this region. Finally, the low concentrations of dissolved forms of N and P recorded during rainfall periods suggest that most of the nutrients flow into the lagoon in particulate form.

The data collected at Station 3 suggest that surface runoff and human activities on the shoreline of the lagoon (bathing, washing dishes and laundry, cassava preparation, etc.) may also contribute to the nutrient enrichment of the lagoon in addition to the discharge of nutrients by the rivers. The watershed of the lagoon in the area where sampling station 3 is located is characterized by a steep slope and recent deforestation that left the soils bare; these two factors are known to enhance runoff and soil erosion [31].

Our data on the temperatures and oxygen concentration variations in the water column also suggest that the internal recycling of allochthonous and autochthonous organic matter may also contribute to the nutrient balance of the lagoon. Temperature gradients were observed in the water column at certain periods/stations, these observations agreed with those typically recorded in shallow tropical lakes [32]. However, our monthly monitoring frequency did not allow us to determine whether the lagoon displays daily stratification or stratification over several days. These temperature gradients were accompanied by decreased oxygen concentrations from the top to the bottom of the water column, and O_2_ concentrations close to zero were found at the bottom the lake at the deepest sampling stations (stations 2 & 4). This finding suggests that releases of SRP may occur in these anoxic areas. The release of phosphorus from sediments is controlled by three main processes: the mineralization of organic matter, desorption, and the reductive dissolution of P bound to iron oxides [e.g., 33–35]. Moreover, the lagoon is very frequently subjected to thermal winds during the warm season, resulting in the formation of waves and Langmuir cells. As shown in another lagoon in the Ivory Coast (Lagoon Ebrié) [36], these processes are involved in the resuspension of sediments in shallow areas, and this effect may also contribute to increasing TP concentrations in the water column as shown by Kristensen *et al*. [37].

We found that the cyanobacterial community of Lagoon Aghien contained several genera that are known to be able to fix atmospheric nitrogen, mainly including *Cylindrospermopsis/Raphidiopsis* and *Dolichospermum*. As the biovolumes of these two genera represented on average 10%±7% of the total phytoplanktonic biovolume of the entire lagoon and over the entire sampling period and represented up to 26% of the total biovolume in December 2017, we can expect that N fixation could contribute to N enrichment in the water, at least in certain periods. However, at the present stage, without any quantification of the nitrogen fixation rate, we cannot obtain any conclusion regarding the contributions of these two genera to the nitrogen cycle.

From our data, it was not possible to estimate the relative impact of all these processes involved in the nutrient loads of the lagoon; however, our data suggest that river inputs and runoff are the main contributors to the nutrient load and especially the phosphorus load. These two processes are mainly associated with rainfall events.

### Dynamics and structure of the phytoplankton community

The highest phytoplankton biomasses were observed during the long dry season, mostly in February and March 2017, while the lowest values were found during the two rainy seasons. Our data also revealed a great temporal variability in the phytoplankton biovolumes, as illustrated by the contrasting data recorded during the two long dry seasons (from December to March) in 2017 and 2018. The only significant difference found among the sampling stations occurred at station 1, where the measured biovolumes were generally higher than those measured throughout the rest of the lagoon. Compared to the other stations, this very shallow station was located close to the mouths of two main tributaries of the lagoon (Bété and Djibi), which together contribute for a large portion of the nutrient fluxes to the lagoon [15]; station 1 was also sheltered from the winds and tidal influence, making it a very quiet area. These characteristics might promote phytoplankton growth and/or accumulation in this area.

No marked seasonality was observed in the structure of the phytoplankton community, as shown by the small temporal variations recorded in the genus richness and diversity (Shannon index) values and in the ß-diversity values. Among the 52 genera found in the lagoon, 16 genera represented >95% of the total biovolume of the phytoplankton community. The variations recorded in the phytoplankton community structure among sampling stations and sampling dates were due to both variations in the relative importances of the dominant genera and the presence/absence of a restricted number of genera with low biomasses. As shown by the CCA results, these variations in the phytoplankton community structure could only be partly explained by the recorded variations in environmental variables, suggesting that other factors and processes are impacting this community and have not yet been identified. The lack of any marked seasonality in the phytoplankton community of Lagoon Aghien might be partially explained by the fact that the taxonomic identification was performed at the genus level, possibly concealing seasonal variations that occur at the species level. Nevertheless, similar absences of seasonality in the phytoplankton communities have already been found in other tropical freshwater ecosystems. For example, the permanent dominance of *C*. *raciborskii* in a small Brazilian reservoir was found to be associated with the high water temperatures recorded throughout the year [38]. In the same way, the perennial dominance of *Botryococcus* and *Peridinium* observed in the phytoplankton community of a tropical reservoir located in l Australia was explained by the potential existence of an equilibrium state in the phytoplankton community when in a stable environment [39]. When looking at the spatial distribution of the different genera, the CCA results showed that on several dates (March 2017, August 2017, and October and November 2017), station 6 was distinguished from the other stations by the high measured biovolumes of the *Aulacoseira* genus, knowing that this station is located close to the output of the lagoon and is under a tidal influence, as shown by Koffy *et al*. [15]. Interestingly, the dominance of this genus in this part of the lagoon was already reported in the first study performed in 2012 by Humbert on the phytoplankton community of this ecosystem [13].

Finally, the analysis of the functional diversity of the phytoplankton community conducted according to the classification method of Reynolds *et al*. [25] showed that all the dominant genera are known to be mostly associated with eutrophic conditions; this result agreed with our measured TP and TN values. The studied phytoplankton community contains a mix of genera displaying various trophic preferences. Indeed, among the dominant genera, phototrophic, phototrophic/N_2_-fixing and mixotrophic genera such as *Peridinium*, *Trachelomonas*, *Euglena* and *Phacus* were found together, with Euglenozoa being very common in shallow lakes in the Ivory Coast [40] and Burkina Faso [41]. All these data suggest that the phytoplankton community is able to use the various sources of nutrients available in Lagoon Aghien (including organic and inorganic nutrients). The main limits of this approach are that (i) the phytoplankton identification at the genus level is a problem for some genera containing species with contrasting habitats and tolerances and (ii) the functional classification proposed by Reynolds *et al*. [25] is mainly based on phytoplankton data collected in temperate areas; thus, cautious should be used when applying this classification method to tropical ecosystems.

### Ecological status of Lagoon Aghien and the implications of its use as a drinking water supply

The chemical and biological variables studied herein indicate that Lagoon Aghien is clearly eutrophic. Indeed, the TP and TN concentrations were always >140 μg L^-1^ and >1.36 mg L^-1^, respectively, the average Chl-a concentrations ranged between 26 and 167 μg L^-1^ and the dominant genera in the phytoplankton community were found to be typical of eutrophic ecosystems. Considering these indicators reported in the papers of Søndegaard *et al*. [42] and Filstrup and Downing [43] for deep and shallow lakes under temperate latitudes, a bad ecological status should be attributed to Lagoon Aghien. Moreover, as shown by Jeppesen *et al*. [44], due to their high water temperatures and the characteristics of their fish communities, subtropical ecosystems are more sensitive than temperate ecosystems to the addition of nutrients, leading to the production of high phytoplankton biomasses.

As a consequence of the eutrophic status of the lagoon, among the ten dominant genera that composed the phytoplanktonic community, five genera (*Cylindrospermopsis/Raphidiopsis*, *Oscillatoria*, *Microcystis*, *Dolichospermum*, *Limnothrix*) are Cyanobacteria, which are known to be potential producers of different cyanotoxins [45]. Such a potential for toxin production, which has already been evidenced in Lagoon Aghien (unpublished data) can constitute a health risk for the local population (i.e., [46]) and make the production of drinking water more difficult, as has been observed, for example, in the water supply of Kampala [47]. In addition, the high quantities of organic matter and the occurrence of potentially toxic cyanobacterial blooms will probably cause the production of drinking water from the lagoon to be costly.

Finally, the sustainability of this water supply is questionable as it is likely that the eutrophication of the lagoon will be amplified in the near future due to the growth of urbanization in the southern part of the watershed and the lack of plans and infrastructure for the collection and treatment of waste.

## Supporting information

S1 Fig Variations in TN concentrations (A) at the six sampling stations, including station 3 (dashed rectangle), which was located close to the shore, and (B) at only the five sampling stations located along the transect (without station 3). The arrows show higher measured TN concentrations at station 3 than those measured at stations 2 and 4. The red squares indicate rainfall peaks.
(PPTX)

S2 Fig Vertical profiles of water temperatures and dissolved oxygen concentrations at the six sampling stations from January 2017 to April 2018. (PPTX)

S3 Fig Boxplot of the temporal variations in the genus richness and diversity (Shannon index) of the phytoplankton community in Lagoon Aghien. (PPTX)

S4 Fig Principal component analysis was performed on the physical and chemical data and Chl-a concentrations collected at the six sampling sites from January 2017 to April 2018 (projection of axes 1 & 2). (PPTX)

S1 Table GPS coordinates and depth of the six sampling stations in Lagoon Aghien. (DOCX)

S2 Table Data used to correct the underestimation of the Chl-a values provided by the YSI probe during the monitoring of Lagoon Aghien. (XLSX)

S3 Table Temperature, turbidity and dissolved oxygen values recorded with the YSI probe at each sampling date and station during the survey. (XLSX)

S4 Table Nutrient concentrations estimated at each sampling date and station during the survey. (XLSX)

S5 Table Biovolume data of each genus estimated at each sampling date and station during the survey. (XLSX)

S6 Table Distribution of the phytoplankton genera observed during the course of the study in Lagoon Aghien. Genus name with blue background = Cyanobacteria; green background = Chlorophyta; light gray background = Dinophyta; yellow background = Euglenozoa; darker grey background = Bacillariophyta.
(XLSX)
