# Supplementary figures and images for: Assessment of some key indicators of the ecological status of an African freshwater lagoon (Lagoon Aghien, Ivory Coast)
Source: PLoS One. 2021 May 6;16(5):e0251065. doi: 10.1371/journal.pone.0251065 (PMC8101731; doi:10.1371/journal.pone.0251065)

## Slide 1
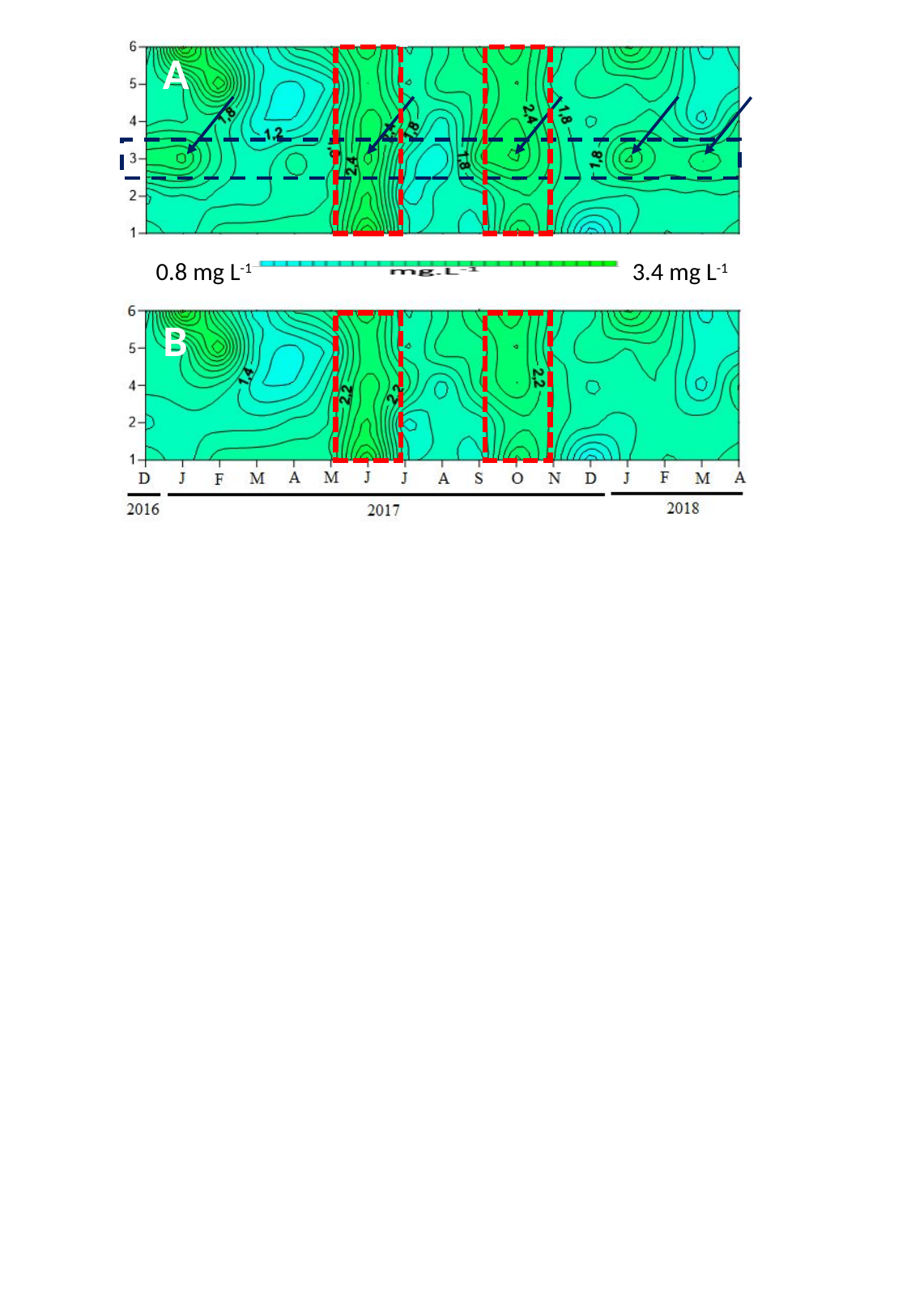

A
3.4 mg L-1
0.8 mg L-1
B

Supplement: S1 Fig — Variations in TN concentrations (A) at the six sampling stations, including station 3 (dashed rectangle), which was located close to the shore, and (B) at only the five sampling stations located along the transect (without station 3). The arrows show higher measured TN concentrations at station 3 than those measured at stations 2 and 4. The red squares indicate rainfall peaks. (PPTX) [file pone.0251065.s001.pptx]

## Slide 1
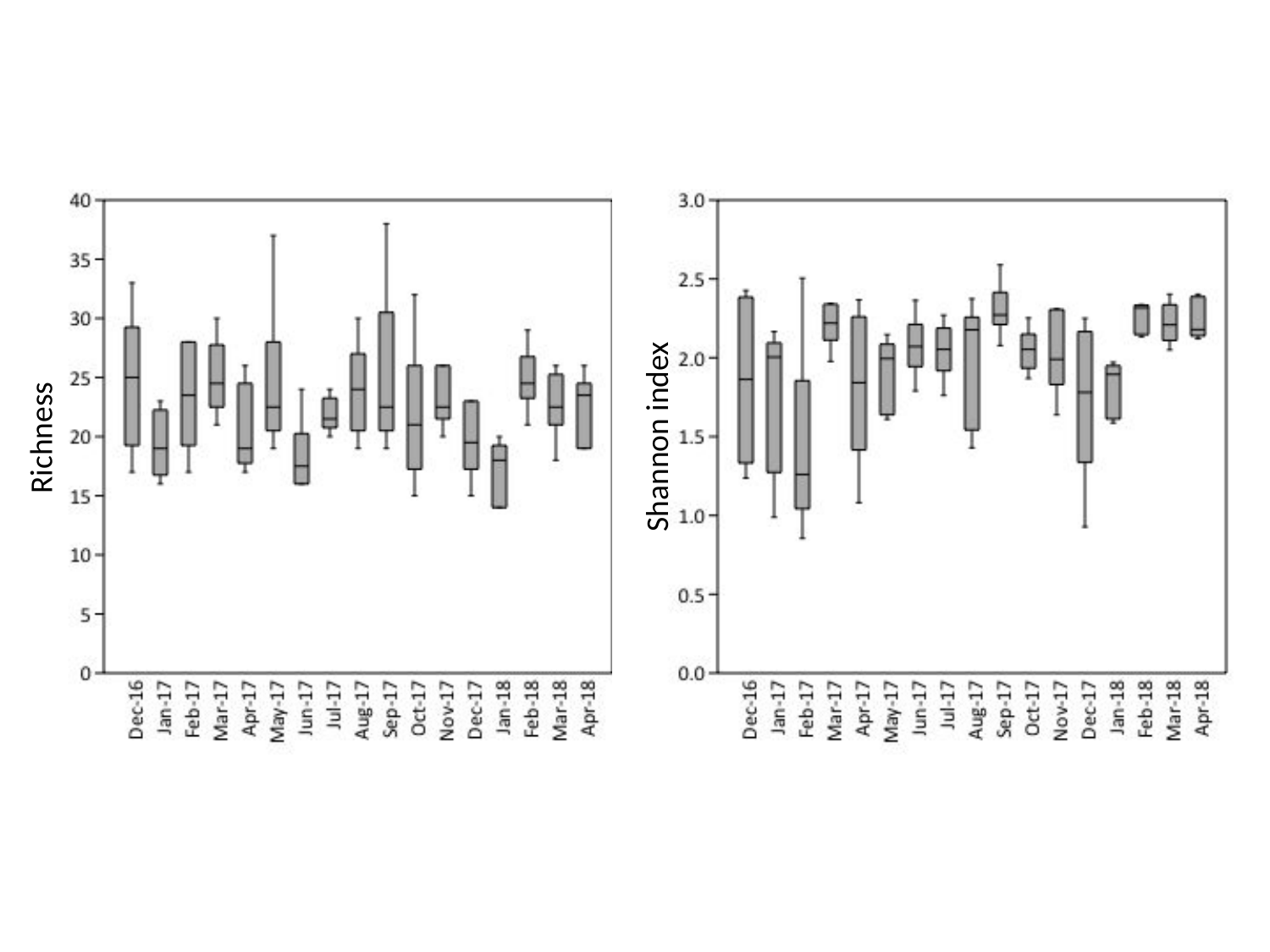

Shannon index
Richness

Supplement: S3 Fig — (PPTX) [file pone.0251065.s003.pptx]

## Slide 1
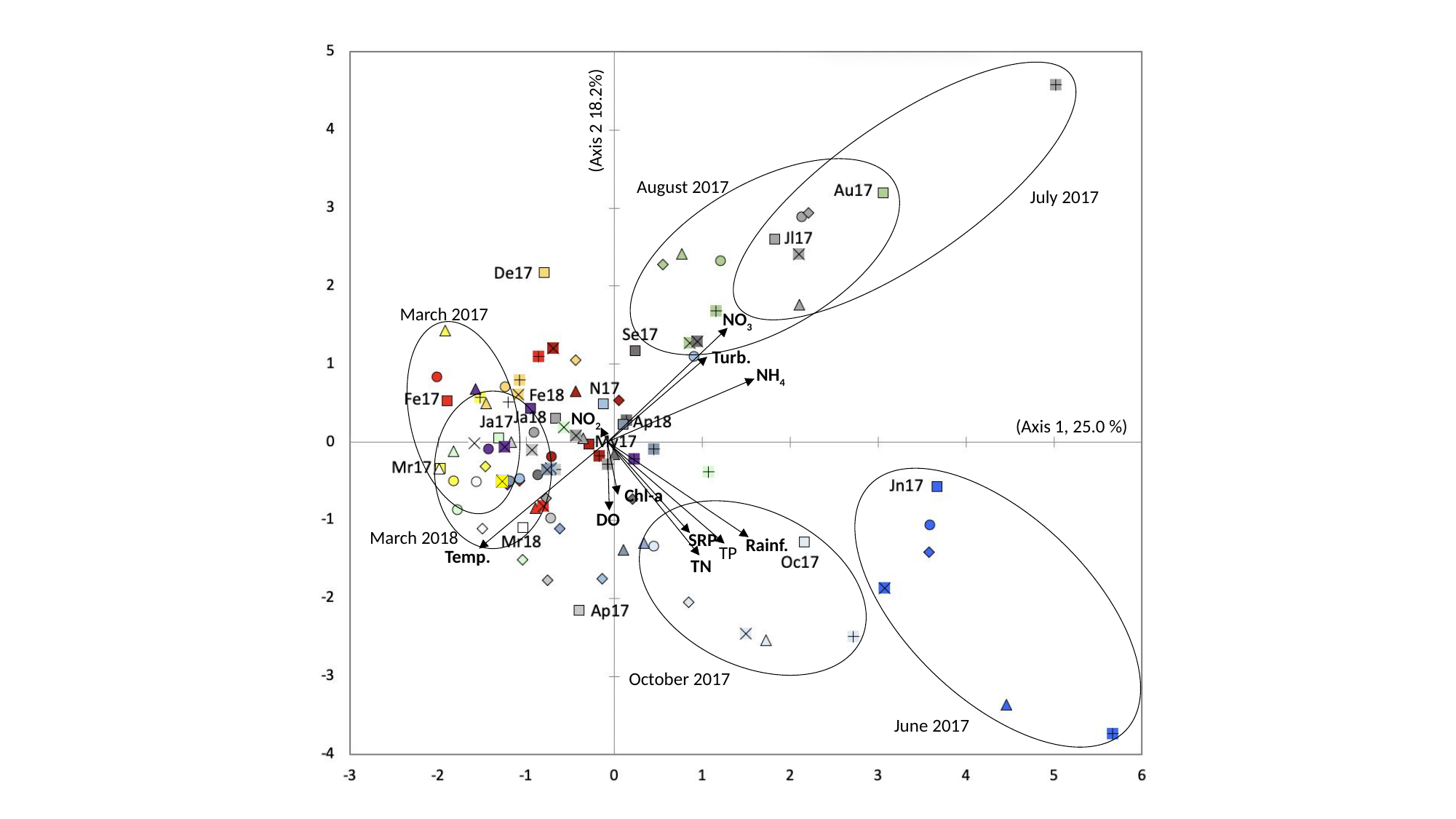

(Axis 2 18.2%)
August 2017
July 2017
March 2017
NO3
Turb.
NH4
NO2
(Axis 1, 25.0 %)
Chl-a
DO
SRP
Rainf.
TP
Temp.
TN
October 2017
June 2017
March 2018

Supplement: S4 Fig — (PPTX) [file pone.0251065.s004.pptx]
