# Supplementary material for: Assessment of some key indicators of the ecological status of an African freshwater lagoon (Lagoon Aghien, Ivory Coast)
Source: PLoS One. 2021 May 6;16(5):e0251065. doi: 10.1371/journal.pone.0251065 (PMC8101731; doi:10.1371/journal.pone.0251065)
Supplement: S2 Fig — (PPTX) [file pone.0251065.s002.pptx]

## Slide 1
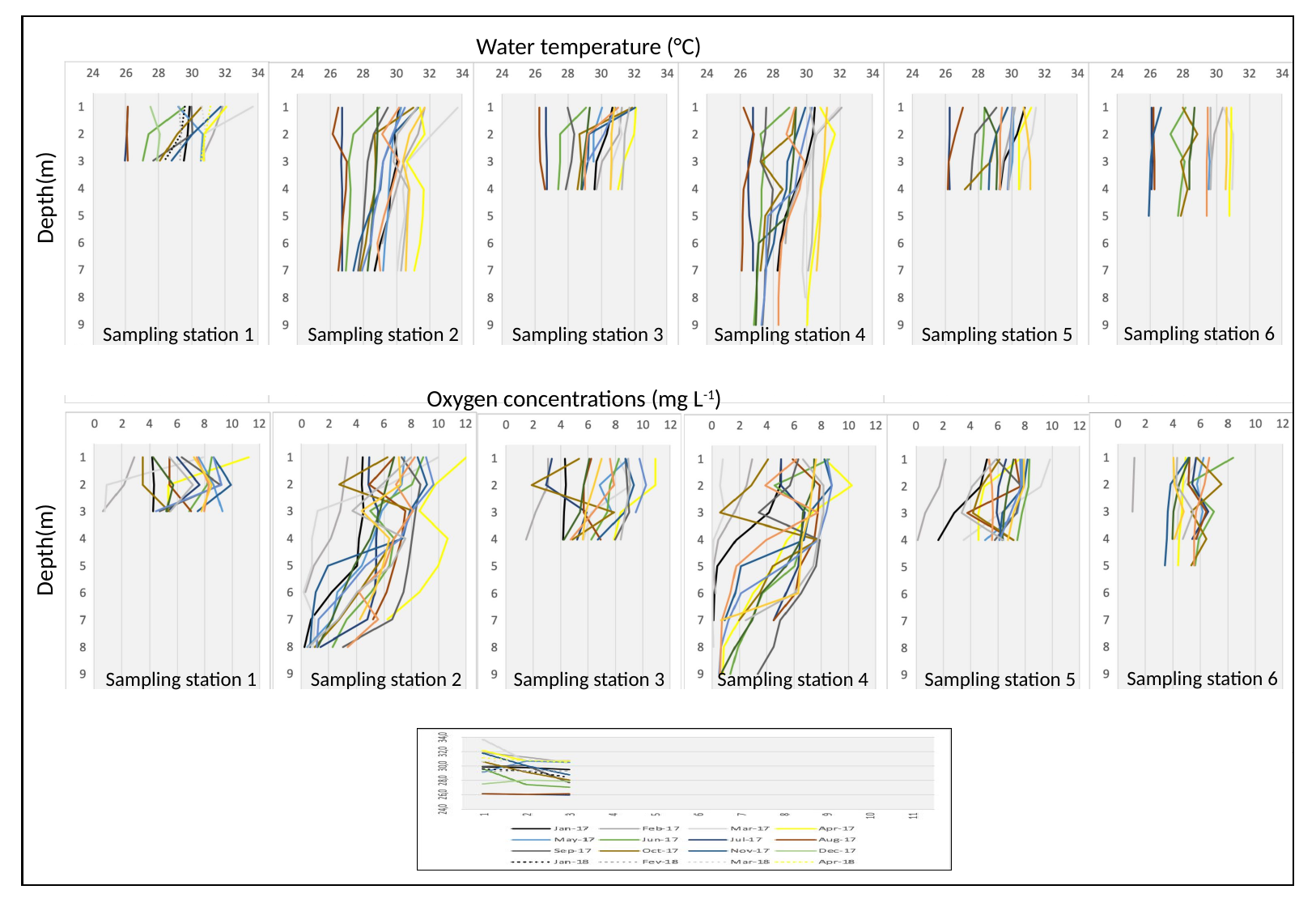

Water temperature (°C)
Depth(m)
Sampling station 6
Sampling station 1
Sampling station 3
Sampling station 4
Sampling station 5
Sampling station 2
Oxygen concentrations (mg L-1)
Depth(m)
Sampling station 6
Sampling station 1
Sampling station 3
Sampling station 4
Sampling station 5
Sampling station 2
