# Supplementary material for: Assessment of some key indicators of the ecological status of an African freshwater lagoon (Lagoon Aghien, Ivory Coast)
Source: PLoS One. 2021 May 6;16(5):e0251065. doi: 10.1371/journal.pone.0251065 (PMC8101731; doi:10.1371/journal.pone.0251065)
Supplement: S1 Table — (DOCX) [file pone.0251065.s005.docx]

|  | **Station 1** | **Station 2** | **Station 3** | **Station 4** | **Station 5** | **Station 6** |
| --- | --- | --- | --- | --- | --- | --- |
| GPS coordinates | N 05°26.173’  W 003°55.246’ | N 05°25.362’  W 003°53.746’ | N 05°25.207  W 003°53.732’ | N 05°24.954’  W 003°52.570’ | N 05°24.183’  W 003°50.913’ | N 05°23.209’  W 003°50.486’ |
| Depth (m) | 3 | 7 | 4 | 9 | 4 | 5 |
